# Ex vivo immunosuppressive effects of mesenchymal stem cells on Crohn’s disease mucosal T cells are largely dependent on indoleamine 2,3-dioxygenase activity and cell-cell contact

**DOI:** 10.1186/s13287-015-0122-1

**Published:** 2015-07-24

**Authors:** Rachele Ciccocioppo, Giuseppina C. Cangemi, Peter Kruzliak, Alessandra Gallia, Elena Betti, Carla Badulli, Miryam Martinetti, Marila Cervio, Alessandro Pecci, Valeria Bozzi, Paolo Dionigi, Livia Visai, Antonella Gurrado, Costanza Alvisi, Cristina Picone, Manuela Monti, Maria E. Bernardo, Paolo Gobbi, Gino R. Corazza

**Affiliations:** Clinica Medica I, Dipartimento di Medicina Interna, Fondazione IRCCS Policlinico San Matteo, Università di Pavia, Piazzale Golgi 19, Pavia, 27100 Italy; Centre for the Study and Cure of Inflammatory Bowel Disease, Clinica Medica I, IRCCS San Matteo Hospital Foundation, University of Pavia, Piazzale Golgi 19, Pavia, 27100 Italy; International Clinical Research Center, St. Anne’s University Hospital and Masaryk University, Pekarska 53, Brno, 656 91 Czech Republic; Servizio di Immunogenetica, Immunoematologia e Medicina Trasfusionale, Fondazione IRCCS Policlinico San Matteo, Università di Pavia, Piazzale Golgi 19, Pavia, 27100 Italy; Clinica Medica III, Dipartimento di Medicina Interna, Fondazione IRCCS Policlinico San Matteo, Università di Pavia, Piazzale Golgi 19, Pavia, 27100 Italy; Chirurgia Generale I, Fondazione IRCCS Policlinico San Matteo, Università di Pavia, Piazzale Golgi 19, Pavia, 27100 Italy; Dipartimento di Medicina Occupazionale, Ergonomia e Disabilità, Laboratorio di Nanotecnologia, Fondazione IRCCS Salvatore Maugeri, Università di Pavia, Via Maugeri 8-10, Pavia, 27100 Italy; Dipartimento di Medicina Molecolare, Centro di Ingegneria Tissutale, INSTM UdR Pavia, Università di Pavia, Pavia, 27100 Italy; Laboratori di Oncoematologia Pediatrica, Fondazione IRCCS Policlinico San Matteo, Piazzale Golgi 19, Pavia, 27100 Italy; Laboratorio di Ematologia, Fondazione IRCCS Policlinico San Matteo, Piazzale Golgi 19, Pavia, 27100 Italy; Centro di Ricerca di Medicina Rigenerativa, Fondazione IRCCS Policlinico San Matteo, Piazzale Golgi 19, Pavia, 27100 Italy; Dipartimento di Onco-Ematologia Pediatrica e Medicina Trasfusionale, Ospedale Pediatrico Bambino Gesù, Via Sant’Onofrio 4, Rome, 00165 Italy

## Abstract

**Introduction:**

Crohn’s disease (CD) is a disabling chronic enteropathy sustained by a harmful T-cell response toward antigens of the gut microbiota in genetically susceptible subjects. Growing evidence highlights the safety and possible efficacy of mesenchymal stem cells (MSCs) as a new therapeutic tool for this condition. Therefore, we aimed to investigate the effects of bone marrow-derived MSCs on pathogenic T cells with a view to clinical application.

**Methods:**

T-cell lines from both inflamed and non-inflamed colonic mucosal specimens of CD patients and from healthy mucosa of control subjects were grown with the antigen muramyl-dipeptide in the absence or presence of donors’ MSCs. The MSC effects were evaluated in terms of T-cell viability, apoptotic rate, proliferative response, immunophenotype, and cytokine profile. The role of the indoleamine 2,3-dioxygenase (IDO) was established by adding a specific inhibitor, the 1-methyl-DL-tryptophan, and by using MSCs transfected with the small interfering RNA (siRNA) targeting IDO. The relevance of cell-cell contact was evaluated by applying transwell membranes.

**Results:**

A significant reduction in both cell viability and proliferative response to muramyl-dipeptide, with simultaneous increase in the apoptotic rate, was found in T cells from both inflamed and non-inflamed CD mucosa when co-cultured with MSCs and was reverted by inhibiting IDO activity and expression. A reduction of the activated CD4^+^CD25^+^ subset and increase of the CD3^+^CD69^+^ population were also observed when T-cell lines from CD mucosa were co-cultured with MSCs. In parallel, an inhibitory effect was evident on the expression of the pro-inflammatory cytokines tumor necrosis factor-α, interferon-γ, interleukin-17A and -21, whereas that of the transforming growth factor-β and interleukin-6 were increased, and production of the tolerogenic molecule soluble HLA-G was high. These latter effects were almost completely eliminated by blocking the IDO, whose activity was upregulated in MSCs co-cultured with CD T cells. The use of a semipermeable membrane partially inhibited the MSC immunosuppressive effects. Finally, hardly any effects of MSCs were observed when T cells obtained from control subjects were used.

**Conclusion:**

MSCs exert potent immunomodulant effects on antigen-specific T cells in CD through a complex paracrine and cell-cell contact-mediated action, which may be exploited for widespread therapeutic use.

**Electronic supplementary material:**

The online version of this article (doi:10.1186/s13287-015-0122-1) contains supplementary material, which is available to authorized users.

## Introduction

Crohn’s disease (CD) is a disabling, chronic inflammatory bowel disease triggered and sustained by a dysregulated immune response toward antigens of the gut microbiota in genetically susceptible individuals [1]. Thanks to the recent strides made in understanding the fine mechanisms responsible for tissue injury, a number of new molecules have been developed and successfully tested in experimental colitis models for therapeutic purposes [2]. However, when used in clinical trials, most of them resulted in disappointing outcomes [3], probably because they were endowed with a single target although the inflammatory response is complex and redundant [2]. This has led to the need for alternative strategies, and cellular therapies, based mainly on the use of stem cells, represent an area of increasing interest thanks to their multi-target action [4]. Among them, mesenchymal stem cells (MSCs) seem to be the best candidate for clinical application by virtue of their easy isolation and ex vivo expansion, their ability to migrate to sites of inflammation where they display potent regenerative function, and their lack of significant immunogenicity, thus allowing them to be infused without the need for preventive immunoablation [5]. Moreover, MSCs have potent immune-regulatory action by virtue of direct cell-cell contact and production of soluble factors, making them particularly attractive for the treatment of immune-mediated diseases [6]. In this regard, the most studied action is that on T cells, where they inhibit both alloantigen- and mitogen-induced proliferation [7], suppress the generation of cytotoxic T lymphocytes [8], and favour the expansion of the regulatory subsets: CD4^+^CD25^+^ transcription factor forkhead box factor (FoxP3)^+^ and interleukin (IL)-10-producing cells [9, 10]. However, there is still much debate on the mechanisms and molecules involved in the immunological action of MSCs [11] because most of the in vitro studies have been carried out by co-culturing MSCs with peripheral blood T cells from healthy subjects [7–10] rather than with T cells isolated from damaged organs of affected patients. Indeed, in recent years, MSCs have been shown to display different behaviour in terms of dampening inflammation and expanding regulatory T-cell populations, depending on the specific disease setting [12]. Consequently, MSCs are not expected to have similar effects in different chronic inflammatory conditions. This prompted us to investigate the mechanisms of immunomodulation of MSCs in CD [13], where no definitive results were obtained when MSCs were applied to treat the luminal [14–16] or fistulising [17–19] forms of CD, although this strategy seems promising and safe [14–20]. The aim of our study, therefore, was to investigate the effects of MSCs on mucosal T-cell lines (TCLs) specific for the muramyl dipeptide (MDP), a motif common to peptidoglican of the bacterial cell wall against which the abnormal immune response is mounted [21], which represents the main effector arm of tissue damage in this pathological condition.

## Methods

### Study population

Twelve patients who had CD (F/M: 8/4, median age of 46.5 years, range of 27–78 years) and who were undergoing both laboratory tests and lower endoscopy as standard workup for their disease [22] were recruited in order to obtain both peripheral blood mononuclear cells (PBMCs) and lamina propria mononuclear cells (LPMCs). Further biological samples were collected from four CD patients (F/M: 1/3, median age of 47 years, range of 28–62) who underwent surgical stricturoplasty or resection for stenosis or both. In all cases, the diagnosis of CD was established according to the widely accepted criteria [22], and the patients’ clinical features are shown in Table 1. Twelve control subjects (F/M: 8/4, median age of 41 years, range of 18–52 years) undergoing peripheral blood harvest and colonoscopy were also enrolled in the study; irritable bowel syndrome was diagnosed in each case, and no patient was taking any medication. The approval by the Fondazione IRCCS Policlinico San Matteo Bio-Ethics Committee was obtained (protocol number 20130002683, administrative procedure number 20130012860, approved on 6 June 2013), and each patient and control gave written informed consent.

**Table 1:** Patients’ clinical features at enrollment

Patient code	Age/Sex	Disease duration, years	Disease localization	CDAI	Therapy
#1	58 years/M	5	Ileo-colonic	170	MESA
#2	27 years/F	2	Ileal	293	MESA, AZA
#3	49 years/F	11	Colonic	253	MESA, AZA
#4	33 years/F	0	Colonic	255	AB, MESA
#5	78 years/M	33	Ileo-colonic	198	MESA
#6	48 years/F	0	Ileo-colonic	274	AB, MESA, PRED
#7	28 years/F	13	Ileo-colonic	426	MESA, BIO
#8	56 years/M	0	Colonic	313	BIO
#9	36 years/F	0,5	Colonic	288	MESA, AZA, PRED
#10	45 years/F	1	Ileo-colonic	301	MESA, AB
#11	49 years/M	0	Colonic	350	BIO-AZA
#12	28 years/F	10	Ileo-colonic	80	MESA
#13	32 years/M	7	Ileo-colonic	320	PRED - MESA
#14	62 years/F	17	Ileo-colonic	274	BIO, AZA
#15	47 years/M	11	Ileo-colonic	216	AZA, AB
#16	28 years/M	3	Ileo-colonic	368	BIO, AZA

*CDAI* Crohn’s disease activity index, *M* male, *MESA* mesalamine, *F* female, *AZA* azathioprine, *AB* antibiotics, *PRED* prednisone, *BIO* biologic (Infliximab)

### Mesenchymal stem cell preparation

Fifty millilitres of bone marrow blood had been harvested from five healthy donors (F/M: 2/3, median age of 35 years, range of 18–61) in order to obtain MSCs for therapeutic purposes [23], and the residual aliquots were used for the present experiments. Briefly, after 48-h culture of bone marrow blood, non-adherent cells were removed and medium was replaced twice a week. Adherent cells were propagated until passage 3, when they were characterized and cryopreserved until use. MSC identification criteria were as follows: spindle-shape morphology, viability of more than 90 %, immunophenotype showing the expression of CD73, CD90, and CD105 surface molecules (>95 %) and the absence of CD34, CD45, and CD31 markers (<5 %), differentiation ability toward adipogenic and osteogenic lineages, and genetic stability. In addition, because the immunomodulatory secretory profile may change among MSC populations obtained from different donors, we used enzyme-linked immunosorbent assay (ELISA) tests to evaluate either the cytokine profile or the human leukocyte antigen (HLA)-G production of each MSC population used and analysed the polymorphisms of the HLA-G exon 8 in the 3′ untranslated region that contains a 14-base pair insertion/deletion by following the method described by Hviid et al. [24]. In this regard, indeed, it has been reported that the insertion of 14-base pair sequence is associated with instability of HLA-G mRNA and lower levels of soluble HLA-G in the serum of healthy individuals [25]. Furthermore, pregnant women homozygous for HLA-G 14-base pair insertion have a significantly lower plasma level of soluble HLA-G than those heterozygous (insertion/deletion) for or lacking (deletion/deletion) the HLA-G 14 base pair [26]. In regard to our MSC donors, four of them were heterozygous (insertion/deletion) for and only one lacked (deletion/deletion) the HLA-G 14 base pair, thus indicating a good capacity to produce the soluble form of this important tolerogenic molecule [27] in all cases.

### Generation of mucosal T-cell lines

Fresh perendoscopic and surgical colonic mucosa specimens were collected from both inflamed and non-inflamed mucosa of patients with CD and from healthy mucosa of control subjects. More precisely, non-inflamed areas were considered those where a normal vascular pattern and an intact mucosa were observed, whereas inflamed areas were those where the normal vascular pattern disappeared and various combinations of the following lesions were evident: edema, erythema, erosions, ulcers, pseudopolyps, granular and spontaneously bleeding zones, and nodular-cobblestone appearance. In accordance with a previously described method [28] that was slightly modified for this experimental setting [29], the samples were immediately rinsed in saline solution and placed in sterile medium: phosphate-buffered saline (PBS) without calcium or magnesium, supplemented with 100 U/ml penicillin, 100 mg/ml streptomycin, and 5 % fetal calf serum. The epithelial layer was then removed by adding 1 mM EDTA and 1 mM dithiothreitol (both from Sigma-Aldrich, St. Louis, MO, USA). After continuous agitation for 1 h at 37 °C, the supernatant was removed and the remaining tissue was digested by collagenase A from *Clostridium hystolyticum* (1 mg/ml; Sigma-Aldrich) in 2 ml of complete medium (Roswell Park Memorial Institute (RPMI) 1640, 10 % fetal bovine serum, and the following antibiotics: 1 % penicillin/streptomycin, 0.25 % gentamicin, and 0.4 % amphotericin B; all from Lonza Group Ltd., Basel, Switzerland) and incubated for 2 h at 37 °C and 5 % CO_2_ by stirring the plate every 15 min. The crude cellular suspension was then passed through a 40-μm filter cell strainer (BD Falcon™; Becton Dickinson, Franklin Lakes, NJ, USA) and centrifuged for 10 min. The cellular population obtained was composed of all the white cells embedded in the mucosa after removal of the epithelial layer, including monocyte macrophages, T and B lymphocytes, natural killer cells, dendritic cells, myofibroblasts, and granulocytes. After further centrifugation, LPMCs were plated in 24-well flat-bottom tissue culture plates (Sarstedt, Newton, NC, USA) at 0.5 × 10^6^ cells per well density in the absence (ctr-TCLs) and presence of allogeneic MSCs (MSC-TCLs) at two different MSC-to-T cell ratios (1:20 and 1:200) and in the presence of 1.5 × 10^6^ autologous γ-irradiated PBMCs (35 Gy, Raycell MK2; Best-Theratronics, Ottawa, ON, Canada) previously obtained by density gradient centrifugation (Ficoll 1.077 g/ml; Amersham Biosciences, Little Chalfont, UK), which were used as antigen-presenting cells, and MDP (10 μg/ml; Sigma-Aldrich) in a final volume of 1.5 ml. The same MSC population was used from the beginning to the end of each experiment, whereas different MSC populations (from a minimum of three donors) were applied in each experimental setting. After 24 h, 40 U/ml IL-2 (R&D Systems, Minneapolis, MN, USA) was added, and thereafter the cultures were fed twice weekly by removing 50 % of the volume and replacing it with fresh medium containing 40 U/ml IL-2. On the 10th day, a second round of antigen + antigen-presenting cells + cytokine stimulation was performed after washing the cellular samples in order to eliminate the residual cells that did not grow in this condition which is specific for T cells. After a further 10 days, TCLs were generated as assessed by flow cytometric analysis (see the following paragraph), showing an almost pure (i.e., >95 %) population of CD3^+^ T cells. An aliquot of each TCL was then harvested from each experimental setting and used to evaluate the effects of MSCs in terms of cell viability (as determined by trypan blue dye exclusion), apoptotic rate, and immunophenotype (as assessed by flow cytometry) as well as proliferative response upon re-stimulation with MDP, as described more in depth in the specific paragraphs below. Finally, supernatants from each ctr-TLC and MSC-TCL at a 1:20 ratio were collected, filtered twice by using 0.2-μm filters to exclude cells, and kept frozen at −20 °C to be used for the analysis of the cytokine profile, soluble HLA-G, and indoleamine 2,3-dioxygenase (IDO) activity (see the specific paragraphs below). All of the experimental conditions with both ctr-TCLs and MSC-TCLs at a 1:20 ratio were also carried out in the absence or presence of 100 μM of 1-methyl-DL-tryptophan (1-MT) (Sigma-Aldrich), a specific inhibitor of the enzyme IDO, and by applying transwell membranes (0.4-μm pore size; Costar; Corning Inc., Corning, NY, USA) where MSCs were pre-plated in the lower compartment to produce an adherent monolayer 18 h before the addition of the T cells in the upper compartment to avoid cell-cell contact.

### T-cell death and immunophenotype evaluation

The rate of T-cell death was evaluated by flow cytometry (FACSCalibur; BD Biosciences, San Jose, CA, USA) with CellQuest Software (Becton Dickinson, Milan, Italy) by using APOPTEST™-FITC (Annexin V-FITC, Propidium Iodide; Nexins Research, Kattendijke, The Netherlands), in accordance with the instructions of the manufacturer, to simultaneously analyse the rates of cells in the early or late phase of apoptosis or dead cells. Furthermore, the surface phenotype analysis was carried out by using three-colour immunostaining (FITC, PerCD, and Cy) with fluorochrome-conjugated antibodies (anti-CD3, -CD4, -CD8, -CD25, and -CD69) and the related isotype control antibodies (all by BD Biosciences Pharmingen, San Diego, CA, USA). The percentage of positive cells was analysed with a gate set on viable mononuclear cells based on their forward scatter/side scatter characteristics.

### Proliferative assay

In a 96-well round-bottom plate (Costar; Corning), cellular aliquots from each established TCL were seeded at a 5 × 10^4^ per well concentration and cultured in the presence of 1 × 10^5^ autologous, γ-irradiated PBMCs (35 Gy; Raycell MK2), and 10 μg/ml MDP as antigenic stimulus or medium alone as negative control or the polyclonal mitogen phytohemagglutinin (PHA 1 μg/ml; Boehringer, Mannheim, Germany) as positive control. Each experimental condition was carried out in triplicate. The plates were then left in a humidified chamber at 37 °C and 5 % CO_2_ for 48 h. Afterwards, T cells were re-suspended and pulsed for 21 h with [^3^H]-thymidine (0.5 μCi per well; GE Healthcare, Buc Cedex, France) and harvested for the evaluation of [^3^H]-thymidine incorporation. The TCL proliferation rate was then assessed in terms of stimulation index (SI) (i.e., the value of counts per minute (cpm) with MDP/cpm with medium alone). A positive response was defined as SI of more than 2.

### Cytokine profile

The levels of interferon-gamma (IFN-γ), tumor necrosis factor-alpha (TNF-α), IL-6, IL-8, and IL-10 were measured in cell-free supernatants by using the Custom Human 5-Plex Array kit (SearchLight; Aushon Biosystems, Billerica, MA, USA), whereas levels of IL-17A were measured with the Human IL-17 Immunoassay (Quantikine ELISA; R&D Systems), those of IL-21 with the Human IL-21 ELISA kit (Cusabio Biotech Co., Ltd., Wuhan Hubei, China), and those of transforming growth factor-beta 1 (TGF-β1) with an ELISA kit (DRG Instruments GmbH, Marburg, Germany) in accordance with the instructions of the manufacturer. Optical density values were measured at 450 nm with a spectrophotometer (Bio-Rad Laboratories Inc., Hercules, CA, USA), and the concentrations of cytokines were calculated according to standard curves and expressed as picograms per millilitre.

### Detection of the soluble human leukocyte antigen-G

The soluble form of the HLA-G was measured by using a standardized ELISA technique with a commercial kit (Exbio/BioVendor, Praha, Czech Republic) in accordance with the instructions of the manufacturer. Briefly, standards and samples were incubated in micro-titration wells coated with a mouse monoclonal anti-HLA-G antibody. After initial washing, mouse monoclonal anti-human β2-microglobulin antibody labelled with horseradish peroxidase was added to the wells and incubated with the immobilized antibody-HLA-G complex. After further washing, the remaining horseradish peroxidase-conjugated antibody was allowed to react with the substrate (H_2_O_2_ with tetramethylbenzidine). The reaction was stopped by the addition of acidic solution, and absorbance of the resulting yellow product was measured with a spectrophotometer at 450 nm. The absorbance was proportional to the concentration of HLA-G. A standard curve was drawn by plotting absorbance values versus the HLA-G concentrations of standards, and the HLA-G levels in the samples were determined by interpolation and expressed as nanograms per millilitre.

### Indoleamine 2,3-dioxygenase activity

IDO activity was indirectly evaluated by quantifying tryptophan and kynurenine concentrations in each TCL supernatant by means of high-performance liquid chromatography by using the SCL-10 VP pump (Shimadzu, Kyoto, Japan) as previously described [30]. L-tryptophan, L-kynurenine, 3-nitro-L-tyrosine, trichloroacetic acid, potassium phosphate, and acetonitrile for the elution buffer (all from Sigma-Aldrich) were of analytical grade. Peak area counts were used to calculate concentrations (EZStart software, version 7.3, Shimadzu, Kyoto, Japan). Tryptophan and kynurenine were referred to nitrotyrosine. The reproducibility of the system was controlled by nitrotyrosine counts, and only variations of less than 5 % were tolerated.

### Small interfering RNA experiment

To knock down IDO, the ON-TARGETplus SMART-pool small interfering RNA (siRNA) (L-010337-01-0005; GE Dharmacon, Lafayette, CO, USA) was used. The sequences were as follows: Human IDO1, NM_002164, sense, 5′-UCACCAAAUCCACGAUCAUUU-3′, antisense, 5′-PUAUGCGAAGAACACUGAAAUU-3′; sense, 5′-UUUCAGUGUUCUUCGCAUAUU-3′, antisense, 5′-PUAUGCGAAGAACACUGAAAUU-3′; sense, 5′-GUAUGAAGGGUUCUGGGAAUU-3′, antisense, 5′-PUUCCCAGAACCCUUCAUACUU-3′; and sense, 5′-GAACGGGACACUUUGCUAAUU-3′, antisense, 5′-PUUAGCAAAGUGUCCCGUUCUU-3′. The ON-TARGETplus siCONTROL Nontargeting Pool (D-001810-10-05; GE Dharmacon) was used as the negative control. siRNAs were transfected into MSCs by the Amaxa Nucleofector II device (Lonza) and the Human MSC Nucleofector kit (Lonza). Briefly, aliquots of 5 × 10^5^ MSCs were re-suspended in 100 μl of the Nucleofector solution in the presence of 1.5 μg of IDO or control siRNA and then electroporated by the U-23 program. Cells were immediately transferred into 37 °C pre-warmed complete culture medium and plated onto six-well dishes. After 72 h from transfection, cells were harvested and lysed for the immunoblotting measurement of IDO levels, while the supernatants were recovered for the assessment of IDO activity and to be used in functional assays.

### Western blot analysis

Whole cell lysates were prepared in an ice-cold immuno-precipitation buffer composed of 10 mM Tris base, 158 mM NaCl, 1 % Triton X-100, 1 % Na deoxycholate, 0.1 % SDS, and 1 Mm EGTA, with the addition of 2 % Protease Inhibitor Cocktail (all by Sigma-Aldrich). The lysates were then centrifuged at 4 °C (10 min, 13,000 revolutions per minute), and the protein concentration of the supernatants was determined by using the Bicinchoninic Acid Kit for Protein Determination (Sigma-Aldrich) at 562 nm. The desired amount of protein (50 μg per lane) was separated by 10 % SDS-PAGE gel (Invitrogen, part of Life Technologies, Carlsbad, CA, USA) and transferred to a 0.2-μm-pore nitrocellulose membrane (Bio-Rad Laboratories Inc.). After 1 h of blocking in 5 % skim milk powder and TBS-T 1× (GE Healthcare and Sigma-Aldrich, respectively), the membrane was incubated with a mouse monoclonal anti-IDO antibody (1:4000; Abcam, Cambridge, UK) overnight at 4 °C. After a 1-h incubation at room temperature with the secondary polyclonal rabbit anti-mouse antibody (1:1000; Dako, Glostrup, Denmark), protein detection was performed by using developing liquids (Kodak, Rochester, NY, USA) maintaining the nitrocellulose membrane in contact with the photographic plate for 10 min. Finally, the blot was stripped and analysed for β-actin as an internal control by using a rabbit anti-human polyclonal anti-β-actin antibody (1:5000; Abcam). Films were acquired by using VersaDoc 3000 (Bio-Rad Laboratories Inc.), and the bands were measured in terms of both surface area and intensity by the QuantityOne software (Bio-Rad Laboratories Inc.) and normalized for β-actin values.

### Time-lapse imaging

To follow the MSC-T cell dynamic interaction, the live cell imaging technique was applied by using an Olympus FluoView FV10i confocal microscope designed for live cell registration (Olympus, Milan, Italy). For this purpose, aliquots of TCLs generated from healthy, non-inflamed CD and inflamed CD mucosa were seeded at 2.5 × 10^5^ cells in 35/10 mm glass-bottom cell culture dishes (Greiner Bio-One, Frickenhausen, Germany) where adherent MSCs had been previously cultured until 80 % confluence. Images of the three conditions used were recorded serially every 30 min for 24 h.

### Statistics

Continuous variables were described as median and 25th-75th percentiles or mean ± standard deviation, whereas categorical variables were expressed as counts and percentages. A preliminary analysis of variance (ANOVA) was used to determine the significance of all the effects and interactions exerted by the tested parameters (cell viability, cell death, cell proliferation, cytokines, and immunophenotype data). A Wilcoxon signed-rank test (for comparisons between two groups) and a Friedman test (for comparisons among three or more groups) were then applied to the biological factors with a statistically significant role at ANOVA in order to obtain both further verification and specific inferencial data from each direct comparison. A paired *t* test with Fisher’s exact test calculated the probability levels of groups with more than four observations. Two-tailed tests were used throughout, and values lower than 0.05 were considered statistically significant [31]. The analyses were performed by using the Stat View 5.5 software package for Macintosh (Abacus Concepts, Berkeley, CA, USA).

## Results

### Mesenchymal stem cell immunosuppressive effects

#### T-cell viability

**Fig. 1:**
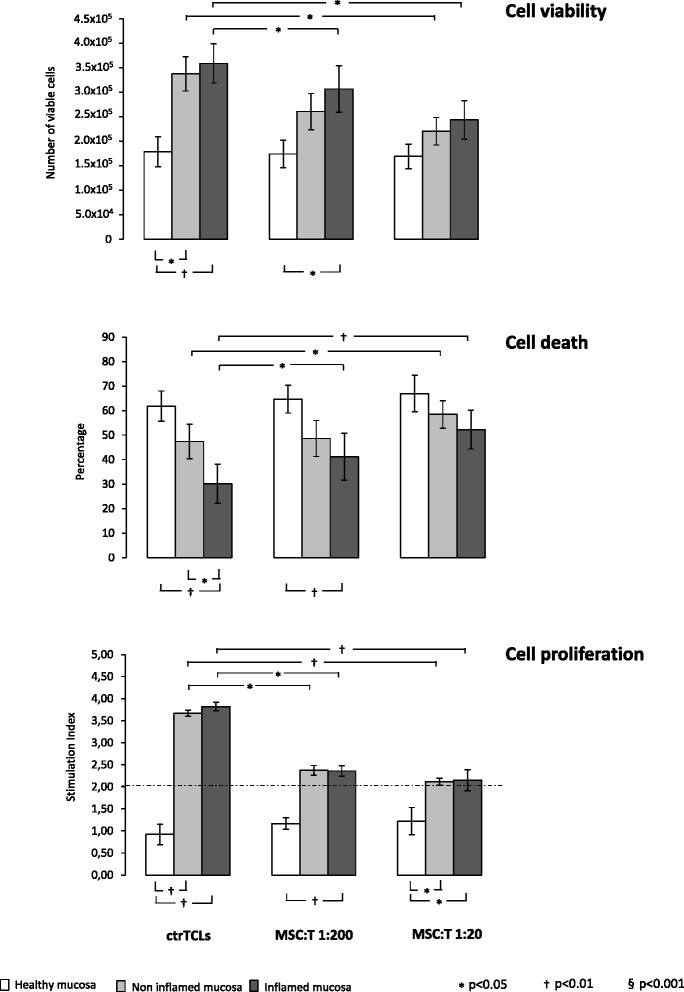
Effects of mesenchymal stem cells on T-cell recovery. The histograms indicate the median values (range) of viable T cells (*upper panel*), their dead rate (*central panel*, where the percentage of cell death represents both the Annexin^+^ and propidium iodide^+^ cells gated on lymphocyte population), and proliferative response (*lower panel*) and refer to at least three separate experiments performed in triplicate in each experimental condition. *White panels* indicate the use of T cells from healthy mucosa, the *light grey panels* indicate the use of T cells from non-inflamed mucosa, and the *dark grey panels* indicate the use of T cells from inflamed mucosa of patients with Crohn’s disease. For comments, see the text. At the preliminary analysis of variance, both the presence and absence of mesenchymal stem cells and the mucosa affected the variability of our experimental parameters (*P* < 0.0001 in all cases). The reported *P* values derive from the non-parametric tests applied to the direct comparisons. *ctr-TCL* control T-cell line, *MSC-T* T-cell lines obtained in the presence of mesenchymal stem cells

Because a T-cell delayed apoptosis has been shown to contribute significantly in sustaining chronic inflammation and tissue injury in CD [29, 32], we explored whether MSCs are able to inhibit T-cell proliferation while inducing cell death in this disease-specific setting. As expected, the number of viable cells recovered from both inflamed and non-inflamed CD mucosa was significantly higher than that obtained when healthy mucosa were used (Fig. 1, upper panel). However, when generating TCLs in the presence of MSCs, a statistically significant reduction of viable cells was observed in those cultures from both inflamed and non-inflamed CD mucosa at 1:20 ratio in comparison with ctr-TCLs, thus approaching the values found in TCLs from healthy mucosa (Fig. 1, upper panel). Moreover, when TCLs from inflamed mucosa were used, this effect was also evident at the lower ratio, although the number of viable cells was still higher than that found when MSC-TCLs from healthy mucosa were used. A specular pattern was found when analysing the apoptotic rate (Fig. 1, central panel), with a lower rate in ctr-TCLs from inflamed CD mucosa in comparison with those from both non-inflamed CD and healthy mucosa, whereas no difference was found when comparing crt-TCLs from non-inflamed CD mucosa with those from healthy mucosa. The incubation with MSCs at a 1:200 ratio caused a slight increase of the apoptotic rate only on TCLs from inflamed mucosa, and values approached those of TCLs from non-inflamed CD mucosa. When the 1:20 ratio was used, the effect was more evident, and MSC-TCLs from both inflamed and non-inflamed CD mucosa displayed values approaching those found in healthy mucosa. Furthermore, in regard to the proliferative response to MDP re-stimulation (Fig. 1, lower panel), no effect was observed on TCLs from healthy mucosa in any condition applied, although they showed a robust response to mitogen stimulus (data not shown). By contrast, when TCLs from both inflamed and non-inflamed CD mucosa were used, a significant proliferative response was clearly evident in a basal condition, which was hampered by MSC incubation at both 1:200 and 1:20 ratios.

#### T-cell immunophenotype

**Fig. 2:**
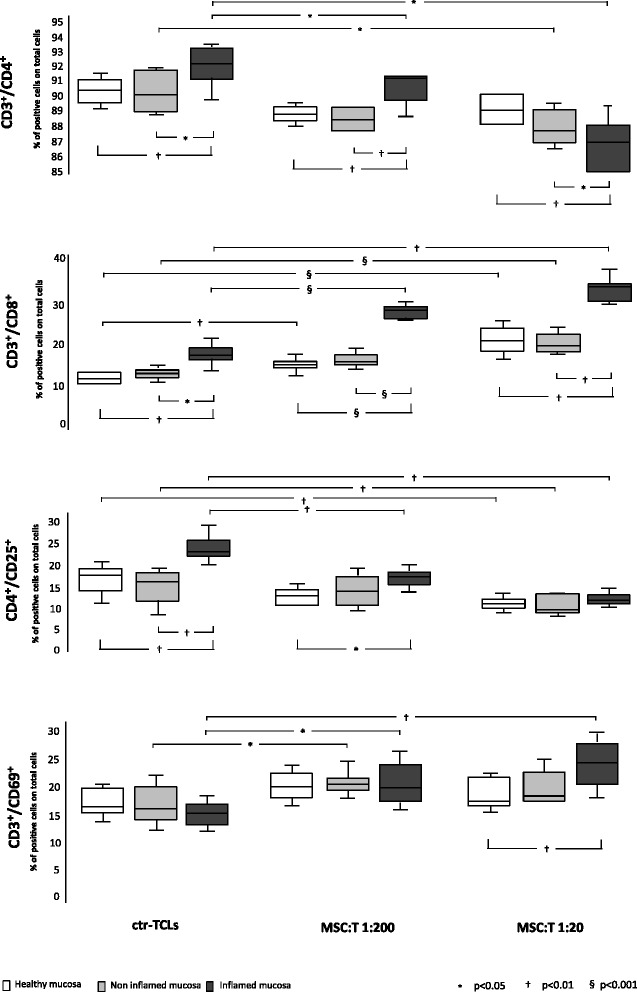
T-cell immunophenotype. The analysis of the expression of surface markers (CD3, CD4, CD8, CD25, and CD69) by T-cell lines generated in the absence (ctr-TCLs) and in the presence of mesenchymal stem cells (MSCs) at two different MSC-to-T cell ratios is shown. All markers were analysed in the gate of lymphocytes as delimited by side scatter-forward scatter parameters, but CD25 in that of CD4^+^ cells. The *white panels* indicate the use of T cells from healthy mucosa, the *light grey panels* indicate the use of T cells from non-inflamed mucosa, and the *dark grey panels* indicate the use of T cells from inflamed mucosa of patients with Crohn’s disease. For comments, see the text. Results are given as median (midline), 25th-75th percentiles (box), and extremes (whiskers) of at least three different T-cell lines for each condition. *P* values are as in Fig. 1. *ctr-TCL* control T-cell line, *MSC-T* T-cell lines obtained in the presence of mesenchymal stem cells

In regard to the immunophenotype (Fig. 2) of ctr-TCLs isolated from inflamed mucosa of patients with CD, a statistically significant upregulation of the activation markers CD4^+^, CD8^+^, and CD4^+^CD25^+^ was found in comparison with ctr-TCLs obtained from both non-inflamed CD and healthy mucosa, whereas the CD69^+^ expression was found to be unchanged. When MSC-TCLs were considered, a ratio-dependent decrease in the frequency of CD4^+^ and CD4^+^CD25^+^ subsets, mirrored by a parallel increase of the CD8^+^ and CD69^+^ populations with respect to ctr-TCLs, was clearly evident when T cells from inflamed CD mucosa were used. A similar, though lesser, effect was found when TCLs isolated from non-inflamed CD mucosa were used, whereas in MSC-TCLs harvested from healthy mucosa of control subjects, only a ratio-dependent increase of CD8^+^ and downregulation of the CD4^+^CD25^+^ subsets were found when the 1:20 ratio in comparison with ctr-TCLs was used. Remarkably, whereas almost all of the CD69^+^ cells co-expressed the CD4^+^ surface marker in ctr-TCLs, a considerable number of them co-expressed the CD8^+^ surface marker when MSC-TCLs from inflamed CD mucosa were used (data not shown). Moreover, mainly in this latter condition, a non-negligible number of CD4^+^ cells also expressed the CD8 molecule, as already found in intestinal mucosa [33] and several autoimmune diseases [34–36]; as a result, the sum of these two subsets exceeded 100 %.

#### Cytokine profile

**Fig. 3:**
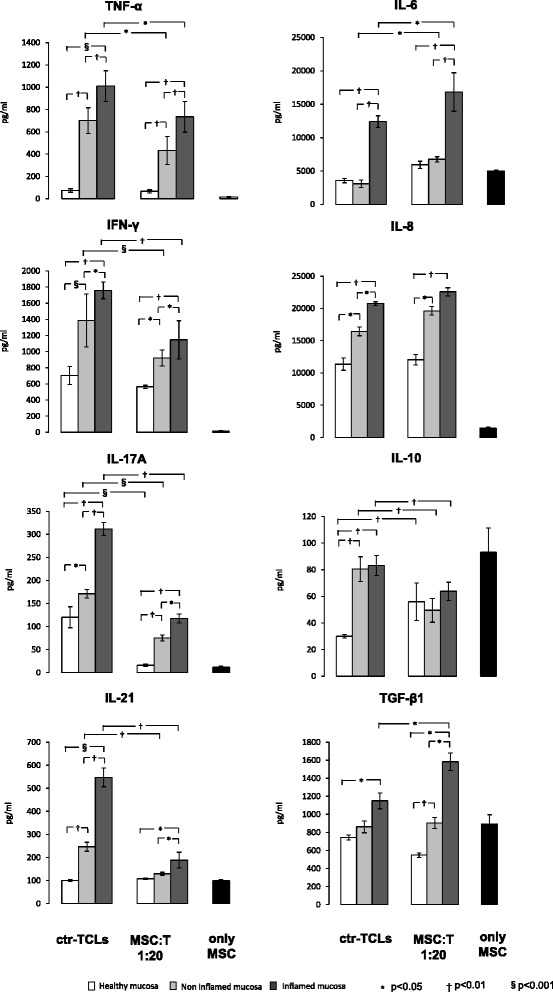
Cytokine profile. The cytokines TNF-α, IFN-γ, IL-6, IL-8, IL-10, IL-17A, IL-21, and TGF-β1 were measured by enzyme-linked immunosorbent assay in cellular-free supernatants of T-cell lines generated in the absence or presence of mesenchymal stem cells (MSCs) at an MSC-to-T cell ratio of 1:20 and of MSCs cultured alone. The measurements were performed in duplicate, and the results are given as median ± standard deviation. The *white panels* indicate the use of T cells from healthy mucosa, the *light grey panels* indicate the use of T cells from non-inflamed mucosa, the *dark grey panels* indicate the use of T cells from inflamed mucosa of patients with Crohn’s disease, and the *black panels* indicate the use of MSC cultured alone. For comments, see the text. *P* values are as in Fig. 1. *ctr-TCL* control T-cell line, *IFN-γ* interferon-gamma, *IL* interleukin, *MSC-T* T-cell lines obtained in the presence of mesenchymal stem cells, *TGF-β1* transforming growth factor-beta 1, *TNF-α* tumor necrosis factor-alpha

When the cytokine profile was analysed (Fig. 3), a statistically significant upregulation of all of the molecules analysed (i.e., TNF-α, IFN-γ, IL-6, IL-8, IL-10, IL-17A, IL-21, and TGF-β1) was found in the supernatants of ctr-TCLs isolated from inflamed CD mucosa in comparison with those obtained from healthy mucosa, whereas a significant increase was observed only for the TNF-α, IFN-γ, IL-8, IL-10, IL-17A, and IL-21 in the supernatants of ctr-TCLs isolated from non-inflamed CD mucosa in comparison with those obtained from healthy mucosa. In addition, an upregulation of TNF-α, IFN-γ, IL-6, IL-8, IL-17A, and IL-21 was shown in ctr-TCLs isolated from inflamed CD mucosa in comparison with the levels found in ctr-TCLs isolated from non-inflamed CD mucosa. When the TCLs were generated in the presence of MSCs, a reduction in the levels of all of the pro-inflammatory cytokines tested (TNF-α, IFN-γ, IL-17A, and IL-21) was invariably found in the supernatants of MSC-TCLs isolated from both inflamed and non-inflamed mucosa of CD patients in comparison with ctr-TCLs, whereas no modification was observed when TCLs from healthy mucosa of control subjects were used; the sole exception is IL-17A, which was almost completely inhibited. Also, when the levels of these cytokines between the supernatants of MSC-TCLs from both inflamed and non-inflamed CD mucosa were compared with those found in MSC-TCLs from healthy mucosa, a strong upregulation is still present; the exception is the IL-21 in non-inflamed mucosa. By contrast, an increase in the pleiotropic cytokine IL-6 was found in the supernatants of MSC-TCLs from both inflamed and non-inflamed CD mucosa in comparison with ctr-TCLs, whereas no modification was observed when TCLs were obtained from control subjects. Surprisingly, the regulatory IL-10 production displayed the opposite pattern since it was lowered by the incubation with MSCs when T cells were isolated from both inflamed and non-inflamed mucosa of CD patients, but it was upregulated when T cells from control subjects were used, thus highlighting the importance of the microenvironment for MSC action. Finally, a significant increase in the other main regulatory cytokine, TGF-β1, was evident only in the supernatants of MSC-TCLs obtained from inflamed CD mucosa in comparison with ctr-TCLs and MSC-TCLs from both non-inflamed CD mucosa and healthy mucosa. No significant modification of the levels of IL-8 was found in MSC-TCLs in comparison with ctr-TCLs in any of the experimental conditions, although the levels were still upregulated in both inflamed and non-inflamed CD mucosa in comparison with healthy mucosa. Remarkably, when MSCs were cultured alone at the same cellular density as that used in the MSC-to-T cell 1:20 ratio (i.e., 250,000 cells per well), they produced high levels of IL-6, IL-8, IL-10, and TGF-β but low levels of IL-17 and IL-21 and undetectable levels of IFN-γ and TNF-α. It is worth noting that the amount of IL-10 was critically lowered when co-culturing MSCs with T cells, thus suggesting that the microenvironment to which MSCs are exposed plays a pivotal role in modulating their secretory profile. This implies that the final concentration of each cytokine found in the MSC-to-T cell co-culture supernatants does not simply represent the sum of the amount produced by each cellular type, but the final milieu resulting from their active interaction. This is why the results were not normalized for the final number of T cells (although the effect of MSCs on T-cell growth might influence the cytokine levels), since an equivalent amount of cells was plated at the set-up of each condition. As far as the soluble HLA-G is concerned, it was abundantly produced in MSC-TCLs co-cultures from both inflamed (19.1 ± 1.9 and 91.3 ± 18.6 ng/ml at 1:200 and 1:20 ratio, respectively) and non-inflamed (11.4 ± 3.7 and 72.2 ± 14.4 ng/ml at 1:200 and 1:20 ratio, respectively) CD mucosa in comparison with MSC-TCLs from healthy mucosa (1.3 ± 0.9 and 3.5 ± 1.6 ng/ml at 1:200 and 1:20 ratio, respectively; *P* < 0.05 for all), whereas undetectable amounts were evident in ctr-TCL cultures. Furthermore, the levels of soluble HLA-G produced by the five MSC populations used in our experiments were very similar (median of 33.1 ng/ml, range of 30.8–35.9).

### Mesenchymal stem cell effects are mostly indoleamine 2,3-dioxygenase-dependent

**Fig. 4:**
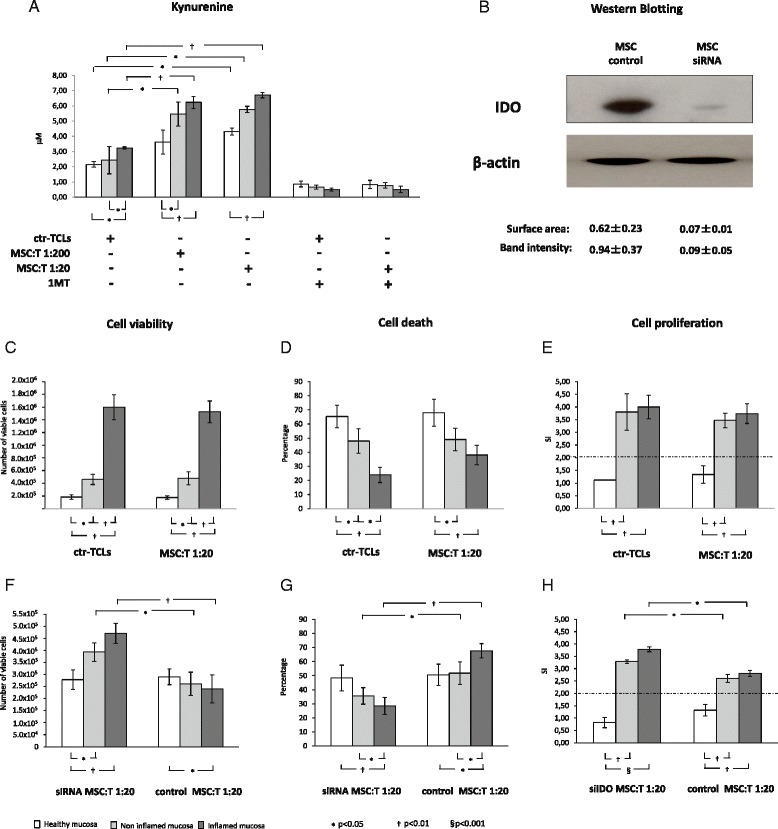
Indoleamine 2,3-dioxygenase (IDO) activity and effects. **a** The histograms indicate the median values (range) of kynurenine concentrations (the *white panels* indicate the use of T cells from healthy mucosa, the *light grey panels* indicate the use of T cells from non-inflamed mucosa, and the *dark grey panels* indicate the use of T cells from inflamed mucosa of patients with Crohn’s disease) of at least three separate experiments performed in triplicate in each experimental condition. **b** Immunoblotting of mesenchymal stem cells (MSCs) transfected with both the siRNA targeting (MSC siRNA) or non-targeting (MSC control) IDO with the mouse monoclonal anti-IDO antibody. The protein levels were measured by scanning densitometry as band area and band intensity, expressed as arbitrary units, and normalized toward β-actin levels. In the *upper part* of the panel, a representative case is shown, and in the *lower part*, the values are given. **c-h** The histograms indicate the median values (range) of viable T cells, their dead rate, and proliferative response in the presence of 1-MT (c-e) and transfected MSC (f-h) and refer to at least three separate experiments performed in triplicate in each experimental condition. *White panels* indicate the use of T cells from healthy mucosa, the *light grey panels* indicate the use of T cells from non-inflamed mucosa, and the *dark grey panels* indicate the use of T cells from inflamed mucosa of patients with Crohn’s disease. For comments, see the text. At the preliminary analysis of variance, both the 1-MT and the use of MSC transfected with siRNA targeting IDO affected the variability of our experimental parameters (*P* < 0.0001 in all cases). The reported *P* values derive from the non-parametric tests applied to the direct comparisons. *ctr-TCL* control T-cell line, *MSC-T* T-cell lines obtained in the presence of mesenchymal stem cell, *1-MT* 1-methyl-DL-tryptophan, *SI* stimulation index, *siIDO siRNA* small interfering RNA

In regard to the IDO activity, as indirectly measured by kynurenine production [37], a statistically significant increase in MSC-TCLs from both inflamed and non-inflamed CD mucosa at either ratio applied was shown in comparison with ctr-TCLs, whereas no effect was observed when MSC-TCLs from healthy mucosa at a 1:200 ratio were used (Fig. 4a). As expected, the incubation with the 1-MT caused a clear drop in the kynurenine levels in all of the experimental conditions tested (*P* < 0.001 for all). By using siRNA targeting IDO, we achieved a knockdown of more than 90 % of its expression in MSCs as measured by Western blot analysis (Fig. 4b). Consequently, the kynurenine production fell to levels of less than 1.0 μM with respect to the amount secreted by non-treated MSCs (3.22 ± 0.1 μM) or MSCs transfected with the non-targeting siRNA control (3.87 ± 0.4 μM). We sought, therefore, to investigate whether the immunosuppressive effects of MSCs were IDO-dependent by adding its specific inhibitor 1-MT or by using the supernatants of MSCs transfected with siRNA targeting IDO.

Interestingly, when the 1-MT was added, an extraordinary increase in the number of viable cells was observed in both ctr-TCLs and MSC-TCLs from inflamed mucosa and, to a lesser extent, even in TCLs from non-inflamed CD mucosa; conversely, no changes were observed in TCLs from healthy mucosa (Fig. 4c). Similarly, the percentage of cell death appeared to be significantly reduced in both ctr-TCLs and MSC-TCLs from CD mucosa in comparison with TCLs from healthy mucosa in both conditions, where no modification was observed (Fig. 4d). Thus, the pro-apoptotic effect of MSCs on CD TCLs (Fig. 1, central panel) was almost completely eliminated. When the proliferative response to MDP re-stimulation was considered, the addition of 1-MT had no effect on TCLs from healthy mucosa and did not modify the proliferation rate of MSC-TCLs from either inflamed or non-inflamed CD mucosa in comparison with ctr-TCLs (Fig. 4e), thus weakening the MSC action (Fig. 1, lower panel). When the influence of the siRNA targeting IDO on MSC effects was explored, a statistically significant increase of cell viability in co-cultures using T cells from CD mucosa was invariably observed in comparison with the co-cultures with T cells from healthy mucosa (Fig. 4f), along with a decrease in the apoptotic rate (Fig. 4g) and restoration of the proliferative response to MDP (Fig. 4h). This did not occur when the supernatants from MSCs transfected with the siRNA non-targeting IDO were used. As far as the immunophenotype profile is concerned, the incubation with 1-MT and the use of MSCs knockdown for IDO did not substantially modify the surface molecule expression of either ctr-TCLs or MSC-TCLs in any of the experimental conditions applied, except for the disappearance of the CD69^+^ subset increase in MSC-TCLs from inflamed CD mucosa (data not shown). Finally, when the IDO inhibitor was added to the MSC-T cell co-cultures, substantial changes, albeit to different extents, in the levels of all of the cytokines tested were clearly observed (Table 2). More specifically, a significant decrease in the TGF-β1, IL-21, and IL-10 production and a strong inhibition of IL-8 and IL-17A expression were observed in both ctr-TCLs and MSC-TCLs isolated from inflamed and non-inflamed CD mucosa, whereas IL-6 appeared to be inhibited in ctr-TCLs from both inflamed and non-inflamed CD mucosa and in the MSC-TCLs from inflamed mucosa. Interestingly, a paradoxical pattern was observed in regard to the IFN-γ levels, which appeared to be reduced by the presence of 1-MT in ctr-TCLs but upregulated in MSC-TCLs in all of the experimental conditions. Also, the TNF-α was similarly decreased in ctr-TCLs from CD mucosa but unchanged in MSC-TCLs from both CD and healthy mucosa. Other than the IFN-γ levels, the addition of 1-MT also substantially reduced the levels of IL-8, IL-10, and IL-21 when using both ctr-TCLs and MSC-TCLs generated from healthy mucosa, where it decreased the levels of IL-6 and IL-17A only in the supernatants of ctr-TCLs and those of TGF-β1 only in MSC-TCLs. Finally, the addition of 1-MT completely eliminated the HLA-G secretion in MSC-TCLs co-cultures from both inflamed and non-inflamed mucosa of patients with CD but did not modify the amount produced by MSC-TCLs from healthy mucosa of control subjects. Again, overlapping results were obtained when the supernatants of MSCs transfected with siRNA targeting IDO were used, in comparison with the use of those of MSCs transfected with the siRNA non-targeting IDO (data not shown).

**Table 2:** Cytokine and HLA-G levels on cell-free supernatants with 1MT

	Condition	TNF-α	IFN-γ	IL-6	IL-8	IL-10	IL-17A	IL-21	TGF-β1	HLA-G
		(pg/ml)	(pg/ml)	(pg/ml)	(pg/ml)	(pg/ml)	(pg/ml)	(pg/ml)	(pg/ml)	(ng/ml)
Healthy mucosa	ctr-TCLs	55.3 ± 20.1	478.9 ± 100.7	409.8 ± 100.3	2005.4 ± 380.7	9.8 ± 2.8	18.9 ± 4.5	45.9 ± 9.5	555.9 ± 74.4	0.2 ± 0.07
		n.s.	p < 0.05	p < 0.05	p < 0.01	p < 0.01	p < 0.001	p < 0.05	n.s.	n.s.
	MSC:T 1:20	70.3 ± 17.9	998.4 ± 209.4	4690.5 ± 220.4	2499.7 ± 277.6	20.8 ± 5.7	18.6 ± 3.5	38.6 ± 9.8	499.2 ± 29.1	1.2 ± 0.4
		n.s.	p < 0.01	n.s.	p < 0.01	p < 0.01	n.s.	p < 0.05	p < 0.05	n.s.
Non inflamed Crohn’s mucosa	ctr-TCLs	290.50 ± 108.4	654.65 ± 88.1	725.73 ± 117.9	2387.0 ± 328.4	11.5 ± 1.8	23.2 ± 2.9	57.5 ± 8.0	517.1 ± 26.9	1.2 ± 0.1
		p < 0.05	p < 0.01	p < 0.05	p < 0.001	p < 0.001	p < 0.001	p < 0.01	p < 0.05	n.s.
	MSC:T 1:20	402.3 ± 109.7	1117.8 ± 156.5	5154.68 ± 855.2	2408.1 ± 389.7	23.8 ± 3.8	23.6 ± 2.7	45.2 ± 4.7	567.4 ± 128.1	1.4 ± 0.3
		n.s.	p < 0.05	n.s.	p < 0.001	p < 0.01	p < 0.05	p < 0.01	p < 0.05	p < 0.01
Inflamed Crohn’s mucosa	ctr-TCLs	428.2 ± 175.8	998.4 ± 328.4	3331.1 ± 538.2	2623.3 ± 475.8	14.1 ± 3.3	36.5 ± 4.2	128.9 ± 9.3	853.2 ± 97.0	1.1 ± 0.3
		p < 0.01	p < 0.01	p < 0.01	p < 0.001	p < 0.001	p < 0.001	p < 0.001	p < 0.05	n.s.
	MSC:T 1:20	827.4 ± 202.1	1290.3 ± 290.5	5468.4 ± 990.3	2318.9 ± 392.1	33.4 ± 7.3	13.2 ± 2.9	127.9 ± 8.3	1071.1 ± 197.7	1.3 ± 0.1
		n.s.	p < 0.05	p < 0.01	p < 0.001	p < 0.01	p < 0.01	p < 0.05	p < 0.05	p < 0.01

Data are expressed as mean ± standard deviation. The *P* values shown refer to the differences between the levels of each molecule on the supernatants of both the ctr-TCLs and MSC-TCLs at MSC-T cell 1:20 ratio in presence of 1-MT with those found in the cultures performed in the absence of 1-MT

*HLA* human leukocyte antigen, *TNF* tumor necrosis factor, *IFN* interferon, *IL* interleukin, *TGF* transforming growth factor, *ctr-TCL* control T-cell line, *n.s.* not significant, *MSC* mesenchymal stem cell, *1-MT* 1-methyl-DL-tryptophan

### Mesenchymal stem cell full action depends on cell-cell contact

**Fig. 5:**
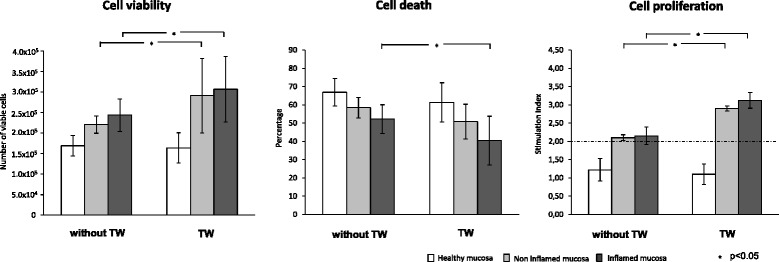
Effects of cell-cell contact on T-cell recovery. The histograms indicate the median values (range) of viable T cells (*left panel*), their dead rate (*central panel*, where the percentage of cell death represents both the Annexin^+^ and propidium iodide^+^ cells gated on lymphocyte population), and proliferative response (*right panel*) and refer to at least three separate experiments performed in triplicate in each experimental condition. *White panels* indicate the use of T cells from healthy mucosa, the *light grey panels* indicate the use of T cells from non-inflamed mucosa, and the *dark grey panels* indicate the use of T cells from inflamed mucosa of patients with Crohn’s disease. For comments, see the text. At the preliminary analysis of variance, the use of the transwells affected the variability of our experimental parameters (*P* < 0.0001 in all cases). The reported *P* values derive from the non-parametric tests applied to the direct comparisons. *TW* transwell

Since close proximity to T cells is needed for MSCs to fulfil their immunological effect [7], we investigated the relevance of cell-cell contact in enabling MSCs to display their action on CD mucosal T cells by using both transwell and live cell imaging techniques. Specifically, by applying a semipermeable membrane that does not permit cell-cell contact, we found that even though the effects of MSCs in terms of reduction of T-cell viability, increase of cell death, and suppression of proliferative response on TCLs from both inflamed and non-inflamed CD mucosa still occurred in such conditions, they were weaker than those observed when cells were permitted to have direct contact, whereas no substantial modification was found when TCLs from healthy mucosa were used (Fig. 5). Similarly, the production of inflammatory cytokines (TNF-α, IFN-γ, IL-17A, and IL-21) from TCLs isolated from both inflamed and non-inflamed CD mucosa was partially restored (ranging from 18 to 36 %) when the transwells were used, whereas that of IL-6, TGF-β1, and especially HLA-G was reduced (ranging from to 21 to 40 %), and the values of IL-8 remained unchanged. Interestingly, the levels of IL-10 increased dramatically in the transwell experiments, thus indicating a powerful interaction of T cells from CD mucosa with MSCs upon cell-cell contact. In this regard, we explored the dynamic of the contact between these two cell populations through the use of live time-lapse imaging, as shown in more detail in the additional movie files (Additional files 1, 2 and 3), where a more intensive interaction was clearly evident when T cells isolated from inflamed mucosa of CD patients were used compared with that found when T cells from non-inflamed CD mucosa or healthy mucosa were used. Moreover, we found that the binding was not stable; in fact, continuous attachment and detachment occurred during the first 6 h, after which a growing number of floating apoptotic figures became evident. Finally, no significant modification of the cytokine levels when TCLs from healthy mucosa were used and of the immunophenotype profile in any experimental conditions was observed by applying the transwells (data not shown).

## Discussion

Following the evidence of efficacy of MSCs in treating experimental colitis [38, 39], several clinical trials where MSCs were applied as a new therapeutic strategy in human CD have been carried out in the last few years [14–20]. The results invariably highlighted the feasibility and safety of their systemic and local infusion in both autologous [14, 17] and allogeneic [15, 16, 19, 20] settings, while efficacy seemed to be optimal when locally injected into fistula tracks [17–19]. However, only patients refractory to standard therapies had been recruited, and, most importantly, no information on the mechanisms by which MSCs exert their action on pathogenic T cells has been available so far [13]. We, therefore, carried out an ex vivo study aimed at evaluating the modifications of mucosal TCLs in terms of cell viability, immunophenotype, and cytokine profile when cultured in the presence of MSCs and at exploring whether they depend on the activity of IDO and cell-cell contact.

### Mesenchymal stem cell immunosuppressive effects

Our results firstly demonstrated that MSCs are able to normalize the T-cell viability in a dose-dependent manner, mainly by increasing the apoptotic rate of TCLs generated from inflamed mucosa. In this regard, a delayed T-cell apoptosis represents a pivotal mechanism in sustaining chronic inflammation in CD [29, 32], and MSCs had been previously proven capable of suppressing activated T-cell functions by inducing a cell cycle arrest at the G_0_-G_1_ phase [40, 41]. Our result of an inhibition of the proliferative response upon antigen stimulation fits with this evidence, and the neutral effect observed when using TCLs from healthy mucosa supports the need for an inflammatory environment to display their full immunomodulatory potential [7, 42]. In this regard, indeed, other than a numeric reduction of the T-cell population isolated from CD mucosa, MSCs also caused a shift of the immunophenotype toward a more tolerogenic pattern and this was due to a significant decrease in the activated CD4^+^CD25^+^ subset and an expansion of the CD69^+^ one. CD69 is an immunoregulatory receptor expressed by early-activated leukocytes at sites of chronic inflammation and by lymphocytes at mucosal surface [43]. It has been demonstrated that a deficiency in CD69 leads to increased production of pro-inflammatory cytokines (such as TNF-α, IFN-γ, and IL-21), reduced FoxP3^+^ regulatory T-cell function, impaired oral tolerance, and more severe colitis [44]. Furthermore, mice lacking CD69 develop an exacerbated form of autoimmune disease, thus indicating a pivotal regulatory role for CD69 in modulating T lymphocyte differentiation through the activation of the Jak-3 signal transducer and activator of the transcription (Stat)-5 signalling pathway, which inhibits T-helper 17 cell differentiation [45]. This evidence provides the basis for the potential of MSCs in dampening inflammation in CD, where an upregulation of this pathway has already been found [46]. Consistent with this notion is the evidence of a statistically significant reduction of the levels of all of the pro-inflammatory cytokines tested (namely TNF-α, IFN-γ, IL-17A, and IL-21) in the supernatants of MSC-TCLs from both inflamed and non-inflamed CD mucosa, together with an upregulation of the immune-regulatory TGF-β1 and the pleiotropic IL-6 molecules. The ability of MSCs to secrete IL-6 upon stimulation with TNF-α and IFN-γ should be considered not only for the interpretation of our data but also in evaluating the inhibition of T-cell response upon antigen stimulation since IL-6 production has been shown to suppress activated T-cell proliferation [47]. Moreover, the levels of two cytokines found abundantly secreted by unstimulated MSCs (i.e., IL-8 and IL-10) appeared unaffected or critically reduced in MSC-TCLs, respectively. In this regard, one would expect a higher concentration of IL-10 in MSC-T cell co-cultures, as found in different experimental conditions where this cytokine proved to be responsible for the immunomodulatory effects of MSCs [48]. However, contrasting results were obtained when measuring the IL-10 levels in culture supernatants of T cells isolated from mice with collagen-induced arthritis re-stimulated with collagen II in vitro since they were found to be either reduced [49] or increased [50], depending on whether the T cells were isolated from mice treated with MSCs or left untreated, respectively. As far as CD is concerned, the relevance of the IL-10 pathway in dampening inflammation was substantiated by the development of severe colitis in infants with mutations of IL-10, IL-10RA, or IL-10RB genes [51] and in mice deficient in IL-10 that underwent spontaneous development of colitis [52]. Unfortunately, the use of human recombinant IL-10 as a new immunomodulatory therapy has been largely disappointing [53] because it induced clinical response in only a small proportion of patients (23.5 %) when used at medium doses (5 μg/kg) while worsening the disease at higher doses (8 μg/kg) because of induction of IFN-γ secretion [54]. Once again, it is likely that peculiar in vivo and in vitro microenvironments differently influence the effect of targeted therapies, while MSCs, though found to lower the levels of IL-10 in vitro, appeared to display a more complex mechanism of action which may be of benefit in blocking the disease-specific pathogenic effector arm and restoring immune tolerance. Our results, indeed, cannot exclude that in the target tissue, IL-10 may contribute to the attenuation of the autoimmune response by MSCs, mainly through induction of regulatory T cells and associated cytokines (i.e., IL-10 and TGF-β).

### Mesenchymal stem cell effects are mostly indoleamine 2,3-dioxygenase-dependent

This enzyme is considered the main actor in inhibiting T-cell growth and function through the degradation of tryptophan, an amino acid essential for lymphocyte proliferation whose depletion induces apoptosis, and the formation of toxic metabolites, such as kynurenine [37]. As far as MSCs are concerned, the IFN-γ gradient produced by locally activated T cells [55, 56] addresses MSCs toward areas of inflammation and induces the expression of IDO [57]. Our findings fully support this evidence because high levels of kynurenine were found in those MSC-TCL co-cultures from CD mucosa that disappeared following the addition of the specific IDO inhibitor, the 1-MT, whereas low levels were observed in co-cultures from healthy mucosa. Furthermore, the presence of discrete amounts of kinurenine also in ctr-TCLs might be explained by the ability of certain immune cell populations to display IDO activity [58], including dendritic cells [59] and regulatory T cells [60]. Nevertheless, our evidence of a full suppression of kynurenine release in MSC-T cell co-cultures through the use of both 1-MT and siRNA knockdown IDO supports the attribution of IDO activity to MSCs in co-culture experiments. Indeed, following the block of either IDO activity or expression, the effects of MSCs on T-cell viability, apoptotic rate, and proliferative response were almost completely reverted when T cells isolated from inflamed CD mucosa were used. In addition, the secretion of most cytokines was significantly reduced, thus indicating that this enzyme contributes greatly to controlling their production, either directly by affecting the number of viable T cells or indirectly by suppressing the production of the master switch molecule for immune tolerance, the soluble HLA-G [61, 62]. This result was partially at variance with the evidence of an upregulation of both surface expression and shedding of HLA-G in dendritic cells when stimulated with both tryptophan and its by-product kinurenine [63]. It is conceivable, therefore, that these two molecules might exert opposite influences on the production of HLA-G by MSCs, with kinurenine acting as a stimulator and tryptophan operating a sort of negative feedback. Taken together, our findings clearly support the notion that IDO activity plays a prominent role in triggering MSC immunological action in this pathological condition.

### Mesenchymal stem cell full action depends on cell-cell contact

It has been found that a cognate interaction between MSCs and allo-stimulated T cells is needed to obtain full HLA-G secretion [62] and immunological function [64, 65]; therefore, we investigated whether cell-cell contact is necessary in order for MSCs to display their effects in our disease-specific setting, by using both transwell and live cell imaging techniques. Our finding of the establishment of more intensive MSC-T cell interaction when T cells isolated from inflamed CD mucosa were used, compared with that observed with T cells from non-inflamed CD mucosa or healthy mucosa, supports this notion. Moreover, at variance with previous evidence showing that activated T lymphocytes bind to MSCs during the first 4 h of culture and then remain trapped for up to 60 h [66], we found that the binding was not stable. In fact, continuous attachment and detachment occurred during the first 6 h, after which a growing number of apoptotic figures became evident (Supplemental files). A possible explanation of this phenomenon is the upregulation of adhesion molecules [67, 68] and chemokines and their receptors [69] on MSC surface following inflammatory stimuli, which enable them to bind to T lymphocytes with high affinity and in a dynamic manner. It is conceivable, therefore, that this mutual interaction enhances IDO activity which, in turn, induces T-cell apoptosis. The involvement of contact-dependent mechanisms was also demonstrated by the use of transwell cultures, which separate MSCs from the responder lymphocytes by a semipermeable membrane. In this condition, although the immunosuppressive impact of MSCs still occurred through the reduction of both T-cell viability and production of inflammatory cytokines, it was much weaker than when cells were allowed to have direct contact. This finding fits with the evidence that both cell-cell contact and secreted molecules are necessary for a stronger suppressive effect of MSCs on T cells [70]. Finally, the fact that, in co-cultures of MSCs and T cells from inflamed CD mucosa where cell contact was not permitted, the levels of TGF-β and IL-10 underwent significant modification compared with those in cell contact conditions underlines the need for close interaction between MSCs and T cells in order to license MSCs to exhibit their full potential in orchestrating the immunological response. In this regard, it has recently been suggested that the expression of both the immune-regulatory molecules TGF-β and HLA-G is modulated in a contact-dependent mechanism [62, 71].

## Conclusions

The results of the present study provide new insights into the immunological mechanisms by which MSCs may dampen the exaggerated inflammatory response of mucosal T cells in CD through the upregulation of IDO activity and HLA-G production. Moreover, to exert their full immunosuppressive potential, MSCs first need to be activated by inflammatory signals and to establish close cell-cell contact with target cells. Our evidence of close, dynamic interaction between MSCs and T cells from CD mucosa suggests that T cells serve as critical feedback signal factors in promoting the immunodulatory action of MSCs and supports the hypothesis that MSCs should be able to deliver their effects to the exact sites of active inflammation. These findings, together with the multipotency, low immunogenicity, amenability to ex vivo expansion, and genetic stability, pave the way for a therapeutic use of MSCs in this pathological condition and not only in refractory cases.

## Additional files

Additional file 1:
**Mesenchymal stem cells with T cells from inflamed Crohn’s mucosa.** Representative time-lapse imaging of a co-culture with bone marrow-derived mesenchymal stem cells of a healthy donor and T cells isolated from inflamed mucosa of a patient with Crohn’s disease. A dynamic and intense interaction is clearly evident mostly during the first 6 h, when the binding between these two cell populations is non-fixed but instead appears as a continuous attachment and detachment, after which a growing number of cells with morphological features of apoptosis (those with nuclear fragmentation and cellular swelling) and apoptotic bodies become evident. The images were serially recorded every 30 min for 12 h. Magnification: 100×. (ZIP 6780 kb) Additional file 2:
**Mesenchymal stem cells with T cells from non-inflamed Crohn’s mucosa.** Representative time-lapse imaging of a co-culture with bone marrow-derived mesenchymal stem cells of a healthy donor and T cells isolated from non-inflamed mucosa of a patient with Crohn’s disease. A slow and continuous interaction is evident, and a few cells with morphological features of apoptosis (those with nuclear fragmentation and cellular swelling) and apoptotic bodies become evident at the end of the period of registration. The images were serially recorded every 30 min for 12 h. Magnification: 100×. (ZIP 6800 kb) Additional file 3:
**Mesenchymal stem cells with T cells from healthy mucosa.** Representative time-lapse imaging of a co-culture with bone marrow-derived mesenchymal stem cells of a healthy donor and T cells isolated from healthy mucosa of a control subject. The absence of any active interaction during the entire period of registration is clearly evident. The images were serially recorded every 30 min for 12 h. Magnification: 100×. (ZIP 9020 kb)

## Abbreviations

1-MT: 1-methyl-DL-tryptophan
CD: Crohn’s disease
cpm: Counts per minute
ctr-TCL: Control T-cell line
DAPI: Diamidino-2-phenylindole
ELISA: Enzyme-linked immunosorbent assay
FoxP3: Transcription factor forkhead box factor
HLA: Human leukocyte antigen
IDO: Indoleamine 2,3-dioxygenase
IFN-γ: Interferon-gamma
IL: Interleukin
LPMC: Lamina propria mononuclear cell
MDP: Muramyl dipeptide
MSC: Mesenchymal stem cell
MSC-TCL: Mesenchymal stem cell T-cell line
PBMC: Peripheral blood mononuclear cell
PBS: Phosphate-buffered saline
PHA: Phytohemagglutinin
SI: Stimulation index
TCL: T-cell line
TGF: Transforming growth factor
TNF: Tumor necrosis factor
